# Genomic Features of Homologous Recombination Deficiency in Breast Cancer: Impact on Testing and Immunotherapy

**DOI:** 10.3390/genes15020162

**Published:** 2024-01-26

**Authors:** Umer Ali, Sunitha Vungarala, Venkataswarup Tiriveedhi

**Affiliations:** 1 Department of Biological Sciences, Tennessee State University, Nashville, TN 37209, USA; uali1@my.tnstate.edu; 2 Meharry-Vanderbilt Alliance, Vanderbilt University Medical Center, Nashville, TN 37209, USA; drsuni.madasu@gmail.com; 3 Department of Pharmacology, Vanderbilt University, Nashville, TN 37209, USA

**Keywords:** genomic instability, homologous recombination defect (HRD), breast cancer, immunotherapy, BRCA 1/2

## Abstract

**Simple Summary:**

Cancer is the second most common cause of death in the USA. Genomic instability is one of the well-established hallmarks of cancer. The homologous recombination repair (HRR) pathway plays a critical role in correcting the double-stranded breaks due to DNA damage. Conventionally, BRCA1/2 genes, which are part of the HRR pathway, have been utilized for testing in breast cancer patients. Nonetheless, other genes in the HRR pathway, such as the RAD51c, PALB2, BRIP1, and BARD1 gene defects, have also been shown to impact breast cancer progression and outcomes. In this communication, we review the impact of the HRR pathway in breast cancer and examine various clinical trials that study the role of HRR genetic testing on treatment outcomes.

**Abstract:**

Genomic instability is one of the well-established hallmarks of cancer. The homologous recombination repair (HRR) pathway plays a critical role in correcting the double-stranded breaks (DSB) due to DNA damage in human cells. Traditionally, the *BRCA1/2* genes in the HRR pathway have been tested for their association with breast cancer. However, defects in the HRR pathway (HRD, also termed ‘BRCAness’), which has up to 50 genes, have been shown to be involved in tumorigenesis and treatment susceptibility to poly-ADP ribose polymerase inhibitors (PARPis), platinum-based chemotherapy, and immune checkpoint inhibitors (ICIs). A reliable consensus on HRD scores is yet to be established. Emerging evidence suggests that only a subset of breast cancer patients benefit from ICI-based immunotherapy. Currently, albeit with limitations, the expression of programmed death-ligand 1 (PDL1) and tumor mutational burden (TMB) are utilized as biomarkers to predict the favorable outcomes of ICI therapy in breast cancer patients. Preclinical studies demonstrate an interplay between the HRR pathway and PDL1 expression. In this review, we outline the current understanding of the role of HRD in genomic instability leading to breast tumorigenesis and delineate outcomes from various clinical trials. Furthermore, we discuss potential strategies for combining HRD-targeted therapy with immunotherapy to achieve the best healthcare outcomes in breast cancer patients.

## 1. Introduction

DNA damage from both extrinsic and intrinsic events can result in pathogenic consequences. One of the DNA damage repair (DDR) mechanisms that is known to have the highest significance in solid organ tumors is the homologous recombination repair (HRR) pathway [1]. This pathway corrects double-stranded DNA breaks (DSB) using homologous chromosomes as templates [2]. Mutations in HRR pathway proteins such as BRCA1/2 are known to induce tumorigenesis [3]. Genomic instability arising from DNA damage is one of the well-established hallmarks of cancer [4,5]. Evidence from the literature suggests that up to 40–70% of triple-negative breast cancers (TNBC) have mutations in HRR pathway genes, a condition referred to as HR deficiency (HRD) [6,7,8,9,10]. In the context of single-stranded DNA breaks, the nuclear enzyme complex, poly (ADP-ribose) polymerase proteins (PARPs) play a vital role in the repair process, thus preventing DNA alterations [11]. There is a positive correlation between the therapeutic efficacy of PARP inhibitors and platinum-based salts in breast cancer patients with mutations in the *BRCA1/2* and HRR genes [12]. Further, mutations in genes associated with the HRR pathway have been shown to impact the therapeutic outcomes of immune checkpoint inhibitors (ICIs) in breast, lung, ovarian, and colon cancer [13].

The ICI-based immunotherapeutic strategies have shown improved overall survival (OS) and progression-free survival (PFS) in breast cancer patients [14]. However, the response rates remain at an abysmal 15–42%, along with a very high tumor relapse rate [15]. Currently, programmed death-ligand 1 (PD-L1) is the only viable biomarker available to predict the success of ICIs [16,17,18]. Several studies have suggested that genomic markers like neoantigen expression, tumor mutational burden (TMB), tumor-infiltrating lymphocytes (TILs), and tumor clonality could have a predictive correlation with the therapeutic success of ICIs [19]. HRD tumors tend to have high somatic mutations and thus increase the overall TMB in cancer cells [20,21]. These somatic mutations lead to the transcription of neo-antigens, which are then presented by professional antigen-presenting cells (APC) to elicit CD8 and CD4 T cell immune responses [22]. Evidence from the literature suggests that a subset of breast cancer patients, specifically, those with HRD, show better outcomes with ICI therapy [23]. In this review article, we discuss the current understanding of the role of HRR mutations in breast tumorigenesis and their potential impact on chemo- and immune-based therapeutic strategies.

## 2. Homologous Recombination Deficiency and Genomic Scars

The DSB is the most deleterious form of DNA damage resulting from a simultaneous break in the phosphate backbones of the two complementary strands [24]. DSBs may be caused by either internal physiologic and metabolic stressors in vivo, or by external events such as ionizing radiation and chemical-induced damage [25,26]. When this damage is not corrected, it might result in genomic instability and eventual tumorigenesis [27]. Various pathways are involved in this repair process. Of these, the homologous recombination repair (HRR) pathway is the major mechanism by which DNA is repaired back to its original configuration [28,29]. 

### 2.1. Homologous Recombination Deficiency (HRD) and BRCAness

**Figure 1:**
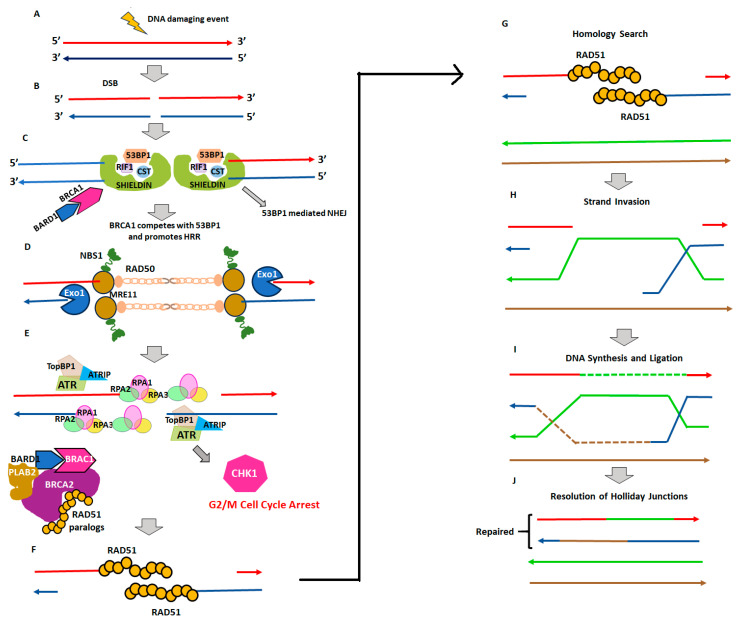
The sequence of steps illustrating the homologous recombination repair pathway. (**A**,**B**) A DNA damage event (ionizing radiation, chemical toxins, etc.) leading to double-stranded DNA break (DSB) first activates p53 binding protein 1 (53BP1) to form a chromatin-protecting protein complex (53BP1-Shieldin-RIF1-CST). (**C**) This 53BP1 complex protects the DNA from further damage and promotes non-homologous end-joining (NHEJ) repair. The NHEJ mechanism is error-prone with a possibility of small insertion or deletion at the site of the DNA lesion. The breast cancer type 1 susceptibility protein (BRCA1) along with its partner BRCA1-associated RING domain protein 1 (BARD1) competes with 53BP1 to preferentially activate the HRR pathway over the NHEJ mechanism. (**D**) The DSB is then sensed by the MRE11 complex (MRE11-RAD50-NBN), composed of the meiotic recombination 11 (MRE11), RAD50, and Nijmegen breakage syndrome 1 (NBS1; also referred to as nibrin), which is then identified by 5′→3′ Exo1 nuclease to start DNA resection on both DNA strands. (**E**) Thus, formed DNA overhang strands are coated by the replication factor A (RPA1–3) complex, which halts further DNA resection. The ataxia telangiectasia-mutated and Rad3-related (ATR) protein kinase localizes to the DNA overhangs along with ATR-interacting protein (ATRIP) to activate topoisomerase DNA II binding protein 1 (TopBP1). This activation switches on the G2/M-phase checkpoint to induce cell cycle arrest and stop further progression of cell division. (**F**) The radiation-sensitive protein 51 (RAD51) now assembles on the DNA overhangs, which is a major rate-limiting step in the HRR pathway mediated by several tumor suppressor proteins, including the BRCA1/ BARD1 complex, BRCA2/PALB2 complex, and RAD51 paralogs. The steps from A to F are referred to as the presynaptic filament formation phase. (**G**) The presynaptic strands undergo a homology search. (**H**) Once the complementary homologous chromosomal DNA strands are identified, the homologous strand invades the RAD51-coated DNA overhang of the presynaptic strand to form a synapse. (**I**) This then leads to DNA replication in the 5′→3′ direction followed by DNA ligation-forming Holliday junctions. (**J**) Finally, the Holliday junctions are resolved to complete the HRR pathway.

This HRR pathway is a multistep process (Figure 1) that involves the recognition of a DSB by an MRN protein complex (consisting of Mre11, Rad50, and Nibrin) and its conversion into single-stranded DNA [30]. This is accompanied by the recruitment of ATM, which phosphorylates proteins such as BRCA1/2 [31,32]. Next, the single-stranded DNA overhang regions are coated by RPA proteins, eventually replaced by RAD51 proteins, which help with identifying the homologous sequence for this single-stranded DNA [33]. BRCA2 mediates the replacement of RPA with RAD51 [34]. This homologous sequence will form the invasion strand [35]. The final step involves the elongation of a single strand by recruitment of complementary DNA bases using an invasion strand followed by ligation and dissolution/resolution of the Holliday junctions, resulting in the complete repair of the DSB [36]. Several germline and constitutional somatic mutations (including methylation of promoter regions) of genes involved in the HRR pathway are associated with many solid organ tumors, such as those of the breast, ovary, pancreas, prostate, and lung [37]. Inappropriate functioning of the HRR pathway (also called homologous recombination deficiency, HRD) leads to genomic instability [38]. In TNBC, reported rates of *BRCA1* mutations range from 10 to 14%, and *BRCA2* mutations from 3 to 5%. Among breast cancers of all phenotypes, *BRCA1* germline mutations were present in 6–8% and somatic mutations in 3–4%; the *BRCA2* germline mutations were in 5–7% and somatic mutations in 2–4% [39,40,41]. Similarly, ‘BRCAness’ is a term often broadly used to refer to an HRD arising from inactivation by mutations or epigenetic modification of other HRR pathway genes (such as *RAD51*, *PALB2*, *BRIP1*, and *BARD1*) [42]. The mutation rate of HRR pathway genes other than germline BRCA1/2 mutations is around 7% among all breast cancers [43] and up to 17% in metastatic breast cancers [44].

**Figure 2:**
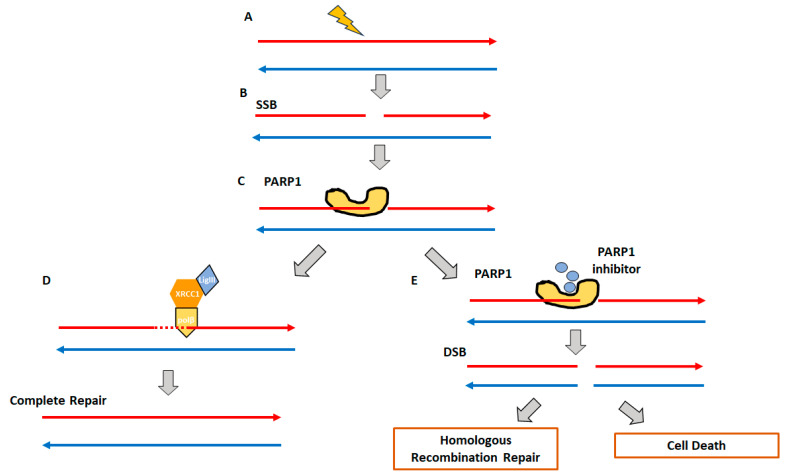
Poly-(ADP-ribose) polymerase (PARP)-based single strand DNA repair. (**A**,**B**) DNA damage event (ionizing radiation, chemical toxins, etc.) leading to single-stranded DNA break (SSB). (**C**) This leads to the activation of the Poly-(ADP-ribose) polymerase (PARP) enzyme complex that mediates the synthesis of a poly-ADP ribose chain. (**D**) The SSB repair is completed by a DNA repair complex (ligase III, polymerase, and XRCC1) through a process called the base excision repair (BER) pathway. (**E**) In the presence of PARP inhibitors (PARPis), the SSB is converted to DSB, which can now either undergo HRR-based correction or, in the presence of HRD, cell death by apoptosis.

In the context of single-strand breaks (SSBs) in DNA, mammalian cells activate an alternate repair pathway (Figure 2). This involves the Poly-(ADP-ribose) polymerase (PARP) enzyme complex, which mediates the synthesis of a poly-ADP ribose chain, which initiates the DNA repair complex (ligase III, polymerase, and XRCC1) through a process called the base excision repair (BER) pathway [45,46]. PARP inhibitors (PARPis) block single-stranded break repair leading to multiple DSB sites, which, when complemented by HRD, will eventually cause cell death [47]. Similarly, platinum-based chemotherapeutic agents bind with purines resulting in DSB [48]. The HRR pathway repairs these platinum-induced DSBs [49]. Therefore, cancer patients with HRD have been considered to have a higher sensitivity to platinum-based chemotherapy [50]. 

### 2.2. Genomic Instability and Genomic Scars

Several constitutional or germline mutations can lead to widespread disruption of genomic stability resulting in tumorigenesis [51]. Identification of these drivers that induce pro-oncogenic pathways will enable the selection of appropriate biomarkers to guide treatment. These pro-oncogenic mutations give a distinct survival advantage to tumors, enabling them to avoid immune elimination and accumulate pro-growth metabolic resources [52,53]. Preclinical studies have shown the anti-tumor efficacy of *BRCA1/2*-deficient breast cell lines, which was reversed following the restoration of *BRCA* genes [54,55]. Alternatively, BRCAness is widely used to refer to the HRD with an apparent lack of germline *BRCA* mutation. In high-grade serous ovarian cancer, there was approximately a 50% prevalence of HRD [56]. This would involve epigenetic and/or genetic inactivation of HRR-associated genes, such as *ATM*, *RAD51*, *PTEN*, *ATR*, and *PALB2*. While PARP inhibitors demonstrated encouraging success in patients with *BRCA1/2* mutation, their limited success in BRCAness was probably due to the misclassification of patients [57]. For example, TNBC and high-grade serous ovarian cancer (HGSOC) have been classified as BRCAness tumor phenotypes, while genomic studies demonstrated that only 35–50% of TNBC and HGSOC have BRCAness [49,58].

**Figure 3:**
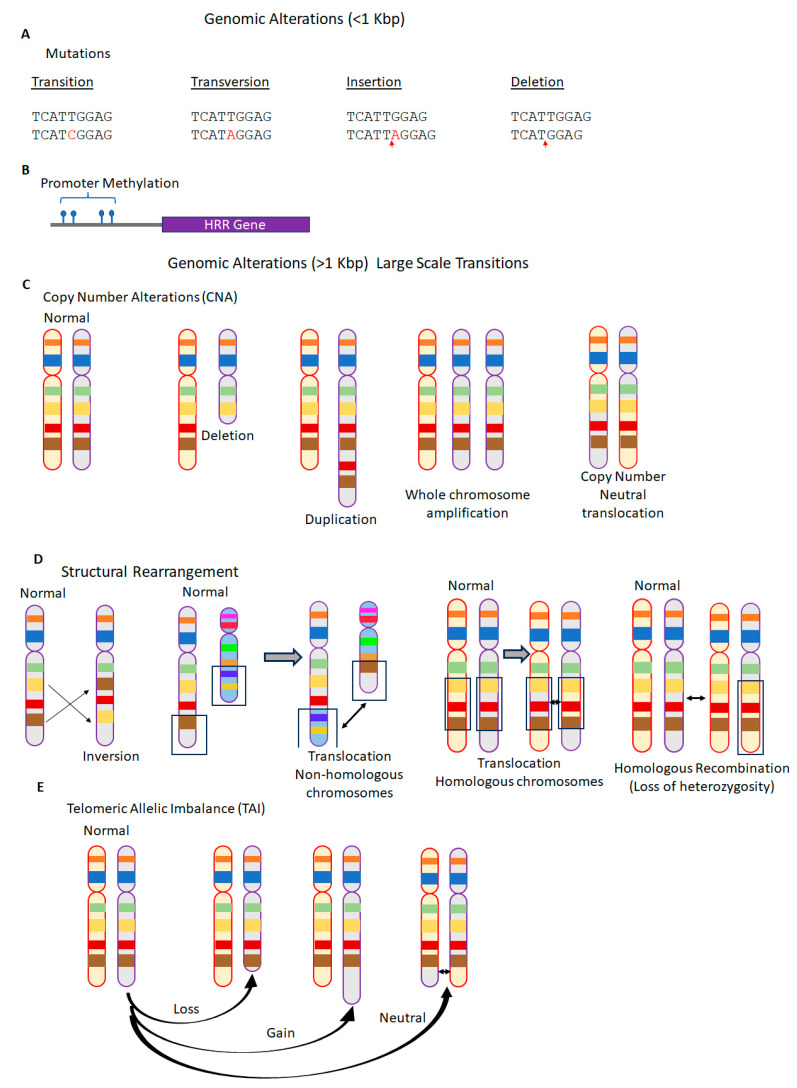
Genomic instability in cancers. (**A**) Genomic alterations involving mutations <1 Kbp in length commonly caused by transition (mutation of a purine to another purine), transversion (mutation of a purine to pyrimidine or vice versa), insertion (addition of a nucleic acid), or deletion (removal of a nucleic acid). Transition and transversion are commonly referred to as substitution-type mutations. (**B**) Genomic alterations involving mutations >1 Kbp in length could also be caused by hypermethylation or demethylation of the CpG islands on the promoter region leading to alterations in gene expression. (**C**–**E**) Genomic alterations involving mutations >1 Kbp in length broadly involve three classes of aberrations, namely, copy-number alterations (CNAs), structural rearrangements, and telomeric imbalance. (**C**) CNA may be a consequence of deletion, duplication, or copy-number-neutral translocation of a portion of the chromosome. (**D**) Structural rearrangement could be due to the rearrangement of a part of the chromosome, a translocation between two non-homologous chromosomes, or a translocation between two homologous chromosomes. Further, structural rearrangement leading to homologous recombination repair could lead to loss of heterozygosity. (**E**) Telomeric imbalance could be caused by the loss, gain, or translocation of telomeres. All these genomic instabilities could have variable effects on the gene expression including impact on final protein length and sequence.

Based on the size and type of variations in DNA sequence, three major classes of genomic scars were shown to arise from HRD (Figure 3), namely, non-structural mutations, structural copy-number alterations (CNAs), and structural rearrangement of DNA sequence [59,60]. Non-structural mutations typically involve one or a few nucleotides (less than 1 Kbp) that include substitutions (transitions/transversions) and indel (insertions, and deletions) mutations. Structural CNA involves either a gain or loss greater than 1 Kbp of DNA sequence on a chromosome, leading to an allelic imbalance between homologous chromosomes. Finally, structural rearrangement refers to either an inversion in the orientation of a DNA sequence within the same chromosome or reciprocal/non-reciprocal translocation between non-homologous chromosomes, which is associated with copy-number-neutral loss of heterozygosity (LOH) [61,62]. Both CNA and copy-number-neutral LOH lead to allelic imbalance causing chromosomal instability [63]. The recent advances in single nucleotide polymorphism (SNP) microarray and next-generation sequencing (NGS) have enabled the accurate measurement of genomic scars [64].

Given the clinical utility of identifying mutations of genes in the HRR pathway, several diagnostic tools have been developed. The TBCRC 048 study suggested that PARPis exerted effective therapeutic efficacy in metastatic breast cancer patients with somatic BRCA mutations and germline PALB2 mutation (an HRR pathway gene) [65]. This study demonstrated the clinical relevance of detecting mutations other than germline BRCA1/2 mutations. Several independent groups have developed tools to measure genomic instability to quantify the impact of HRD in solid organ tumors [66,67]. These tools include large-scale state transition (LST, >10 Mb large breaks in chromosomal regions), telomeric allelic imbalance (TAI, an allelic imbalance due to the loss or gain of the subtelomeric region), and loss of heterozygosity (LOH, loss of >15 Mb regions of heterozygosity) [68,69]. Popova et al. showed that HRD is strongly associated with LST in TNBC, suggesting the potential application of LST as a genomic scar marker [70]. Similarly, Timm et al. have shown that HRD is associated with all three genomic scar markers (namely, LST, TAI, and LOH) [71]. This led clinical genetics laboratories to develop the HRD score, which could be used to predict the therapeutic success of PARPis and platinum-based chemotherapy [72]. 

Recently, next-generation sequencing (NGS) has been widely used to check the specific HRD mutational signatures in solid organ tumors [73]. The HRD score is a bioinformatics-based calculation of whole genome analysis as an unweighted numeric sum of LOH, TAI, and LST. The DNA obtained from tumor tissue and normal tissue will be utilized to perform next-generation sequencing (NGS)-based assays to generate genome-wide custom enrichment single nucleotide polymorphism (SNP) profiles with up to 54,000 targets. This panel also includes 685 probes that target BRCA1/2 coding regions. Timm et al. have extensively discussed this panel design process [71]. The LOH component of the HRD score is calculated based on the number of LOH regions longer than 15 Mbp but shorter than the whole chromosome [74]. The TAI component of the HRD score is calculated based on the number of allelic imbalance regions that are longer than 11 Mbp and extend to the subtelomeric region but do not cross the centromere [75]. The LST component of the HRD score is defined as the number of breakpoints between regions longer than 10 Mb after filtering out regions shorter than 3 Mb [70]. The LST score is proportional to the ploidy of tumor samples. Several R-program-based bioinformatics/statistical tools have been utilized in calculating HRD scores. A high HRD score of 42 was used based on the 5th percentile cut-off in BRCA1/2-deficient breast and ovarian tumors [10]. In the future, a combination of whole genome sequencing and HRD score could be used as a more sensitive and specific tool to predict the efficacy of various therapeutic regimens. In addition to genomic testing, functional immunofluorescence and immunohistochemistry tools, such as nuclear accumulation of RAD 51, are also being evaluated as viable testing options to check for HRD in ovarian, prostrate, TNBC, and other solid organ tumors [76,77,78].

A major challenge exists in identifying HRD status in tumors other than regular germline *BRCA1/2* mutations [79]. Comprehensive HRD scores have been reported by several clinical testing agencies. FDA-approved clinical genetics testing to identify mutations in HRR pathway genes is offered by Myriad’s BRCA Analysis CDx [80,81]. This testing tool was utilized in a phase II clinical trial (NCT02401347) to determine mutations in HRR pathway genes and determine the efficacy of PARPis [82]. Similarly, the Foundation One CDx assay by Foundation Medicine is also an FDA-approved clinical genetics assay on tumor tissue to determine microsatellite instability and tumor mutational burden [83]. The results from the clinical trial (NCT03367689) using this assay are still pending [84]. Major findings from two big randomized trials in ovarian cancer, ENGOT-OV16/NOVA and ARIEL3, suggest that HRD status is a good predictor of the potential therapeutic efficacy of PARPis but has a limited role in identifying resistant tumors [50,85]. Another major limitation of these clinical testing tools is their inability to test all genes involved in the HRR pathway. An alternative clinical testing approach for the identification of HRD status is mutational signature 3 (Sig3 or SigMA), which utilizes limited sequencing data derived from gene-focused panel sequencing [86]. Two clinical trials, NCT01623349 and NCT02624973, have utilized these tools to predict the therapeutic efficacy of PARPis [87,88]. However, more prospective clinical trials are required to validate this SigMA assay.

## 3. Impact of BRCAness on Tumor Immunity

**Figure 4:**
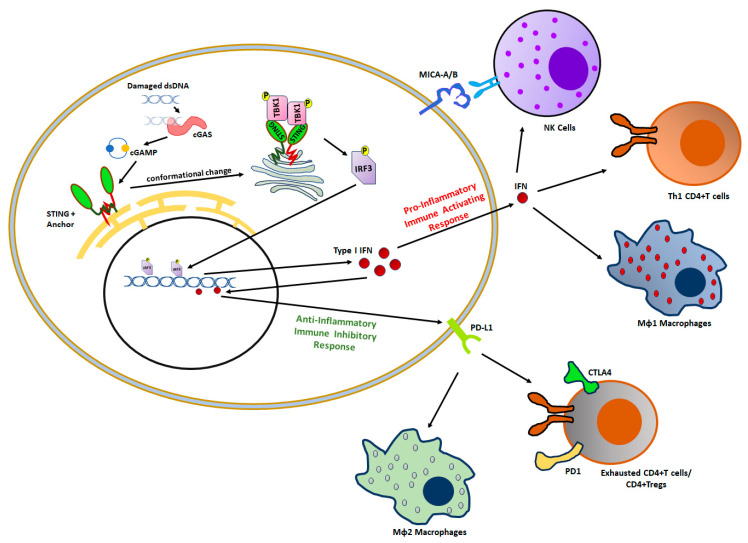
DNA damage response (DDR) induces the stimulator of interferon genes (STING) pathway. Following DNA damage there is an increase in cytosolic DNA breakdown fragments which induce cyclic guanosine monophosphate–adenosine monophosphate synthetase (cGAS) leading to the synthesis of the second messenger, 2′3′-Cyclic GMP-AMP (cGAMP). The STING adapter protein, otherwise anchored on the endoplasmic reticulum (ER), upon binding with cGAMP causes a conformational change in the STING protein. This causes activation of the IRF3 transcription factor which then relocalizes to the nucleus leading to the expression of inflammatory type I interferon (IFN) cytokines. The IFNs have an antithetical impact. On one hand, they activate anti-tumor NK cells, Th1/CD4 + T cells, and MΦ1 macrophages. On the other hand, IFNs also lead to the transcription of immune-exhaustion factors, like programmed death ligand 1 (PD-L1), leading to activation of pro-tumor MΦ2 macrophages, FoxP3 + regulatory T cells (Treg), and exhausted PD1 + CD4 + T cells.

Tumor-infiltrating immune cells (TIIC) can exert a complex, apparently opposite, pro-tumor or anti-tumor response [89]. This contrasting phenomenon is dependent upon the specific phenotype of the immune cells within the tumor. Both innate and adaptive immune cells play a role in these antithetical responses. Altered DNA damage responses (DDRs) following constitutional mutations of the genes involved in the HRR pathway or exposure to cytotoxic agents cause activation of the inflammatory STING (stimulating interferon gene) pathway leading to anti-tumor response (Figure 4) [90]. The soluble DNA released from defective DDR will cause an enzymatic activation of cyclic guanosine monophosphate–adenosine monophosphate synthetase (cGAS) leading to the synthesis of the second messenger, 2′3′-Cyclic GMP-AMP (cGAMP) [91]. This, in turn, upon binding with the STING adapter protein anchored on the endoplasmic reticulum (ER) causes a conformational change in the STING protein. The conformational change induces the migration of STING from the ER to the Golgi apparatus, which is associated with the recruitment of phosphorylated TBK1 and IKK kinase. These, in turn, activate downstream IRF3 and NF-kB, causing a robust secretion of inflammatory innate immune type I interferon (IFN) cytokines resulting in an anti-tumor response [92]. In the context of the tumor microenvironment (TME), activation of STING pathways in antigen-presenting cells (APCs) enhances the tumor-associated antigen-specific antitumor CD4 and CD8 T cell responses [93]. Interestingly, damaged DNA can also activate an ‘alternate STING pathway’ through ATM-TRAF6 signaling mediated by protumor cytokines TGFβ and IL-6. Further, this alternate STING pathway is shown to enhance tumor cell expression of immune inhibitory molecules, such as PD-L1, resulting in a pro-tumor response [94]. In line with this, preclinical studies by Pellegrino et al. have shown that treatment with olaparib upregulated tumor expression of PD-L1 [95]. Also, this led to the hypothesis that a combination of DNA-damaging drugs (such as platinum-based therapy and PARPis) with ICIs could lead to better therapeutic outcomes in cancer patients. Molecular studies from the IMpassion130 trial showed that the combination of nab-paclitaxel with ICIs in TNBC patients who specifically overexpressed PD-L1 on immune cells improved therapeutic outcomes, thus suggesting that DNA-damaging drugs sensitize tumors to ICI therapy [96].

### 3.1. Tumor-Infiltrating Innate Immune Cells

Natural killer (NK) cells are the major anti-tumor innate immune cells. Low MHC class I expression is a well-established immune escape strategy by tumor cells which can be efficiently countered by the cytotoxic effector impact of NK cells. These innate immune cells are commonly identified by the surface marker CD3^−^CD56^+^ and are further divided into CD56^bright^ and CD56^dim^ subgroups [97]. There is a phenotypic difference between circulating NK cells and tissue-resident NK cells. Distinct cell-differentiating processes play a role in the terminal differentiation of NK cells in specific tissues. In the context of the tumor microenvironment, NK cell detection of deviant cells is dependent upon IL-12, IL-15, and IL-18 cytokine signaling, trans-presentation by dendritic cells, the balance between activating and inhibitory signals, and interaction with MHC-I on the surface of target cells [98]. The specific role of NK cells seems to be different among various cancer types. In the CIBERSORT analysis, NK cells were divided into resting and activated subtypes, each contributing to the formation of the tumor microenvironment [99]. NK cells, due to their expression of CD16, a low-affinity Fc fragment of IgG receptor, are involved in antibody-dependent cellular cytotoxicity, which is critical in the clinical application of monoclonal antibody-based immunotherapy. The CD3^−^CD16^hi^CD56^dim^ NK cells have a higher cytotoxic effect due to their ability to release granzyme and perforin upon recognition of MICA and MICB ligands (MHC class I polypeptide-related sequence A and B) that are usually expressed by cells undergoing inflammatory stress. On the other hand, the CD3^−^CD16^lo^CD56^bright^ NK cells have poor cytotoxic activity. When found in the tumor microenvironment, they play a critical role in the release of chemo-attractive cytokines resulting in the tumor infiltration of other adaptive (such as CD8 and CD4 T cells) immune cells [100]. Although CD3^−^CD16^lo^CD56^bright^ NK cells reflect different clinical outcomes in different cancers, the co-expression of other functional molecules on NK cells, including NKp30^+^ and NKp46^+^, indicates favorable survival [101]. This highlights an important fact, which is that full activation and not just infiltration density determines the final NK-cell-associated anti-tumor response. Interestingly, the CD56^dim^ cells have been shown to express PD1 in solid organ tumors and are therefore inactivated upon binding with its ligand PD-L1 on cancer cells [102]. The DNA-damage-mediated activation of the STING signaling pathway has been shown to promote NK cell anti-tumor impact. Based on this, various preclinical studies are underway to determine the anti-tumor cytotoxic impact of NK cells following treatment with STING agonist drugs [103]. 

Another innate immune cell phenotype, MΦ2 macrophage, is associated with tumor progression and metastasis. The IL-4, IL-10, and IL-13 cytokines released from CD4+Th2 immune cells have been shown to induce a phenotypic switch of naïve MΦ0 macrophages to the pro-tumor MΦ2 phenotype [104]. However, there seems to be considerable debate in the literature regarding the correlation between tumor grade and the frequency of tumor-infiltrating MΦ2 macrophages. This is because of the specific marker used to identify the phenotype of tumor-associated macrophages (TAMs). The expression of CD68^+^ TAMs seems to correlate with a negative prognosis of breast cancer, along with an unfavorable positive correlation with tumor size, grade, lymph node metastasis, and vascular invasion. However, the expression of CD68 as a viable marker for TAMs has raised some doubts [105]. Given these concerns, another TAM marker, CD163, was utilized. Multiple studies on TNBC, prostate cancer, and colorectal cancer have shown that CD163 expression is a negative prognostic marker [106]. Interestingly, MΦ2 macrophages are associated with anti-inflammatory responses such as wound healing and repair. This has led to research exploring the potential role of MΦ2 macrophages in resolving DNA damage repair mechanisms. 

### 3.2. Tumor-Infiltrating Adaptive Immune Cells

Lymphocytes (T and B cells) constitute the adaptive immune arm of tumor-infiltrating immune cells and have been shown to be localized either as nests in direct cell-to-cell contact with tumor cells or in the stroma component of tumors. Lymphocytes in the tumor-draining lymph nodes also play a critical role in both pro- and anti-tumor responses. A high frequency of tumor-infiltrating lymphocytes (TILs) has been shown to be associated with better prognosis in various breast cancer subtypes [107]. The BIG-02-98 trial, which analyzed a cohort of 256 node-positive TNBC patients, found a 15–17% and 17–27% reduction in recurrence risk with every 10% increase in stromal TILs and intratumoral TILs, respectively [108]. Similarly, findings from 506 tumor tissues analyzed from two big phase III trials, the Eastern Cooperative Oncology Group (ECOG) E2197 and E1199 trials, demonstrated that a 10% increase in stromal TILs is associated with statistically significant disease-free survival (DFS) [109]. However, the exact role of TILs in tumorigenesis is still debated. Among TILs, the effector immune cells (CD8+T cells and Th1/CD4 + T cells) are associated with tumor immune elimination, while inhibitory phenotypes, such as CD4 + FoxP3 + T cells (Treg), are associated with tumor progression. The secretory cytokine profile from these various TIL phenotypes is different [110]. However, as the impact of these cytokines is within the tumor microenvironment, serum cytokine analysis is not helpful in predicting the specific TIL phenotype frequency. 

Based on the findings from the IMpassion 130 trial, anti-PDL1 monoclonal antibody (mAb)-based therapy is approved as the first-line agent in TNBC treatment along with nab-paclitaxel [96,111,112]. Furthermore, this study formed the basis for the FDA approval of clinical testing on tumor tissues to assess PD-L1 expression by SP142 assay to determine the applicability of anti-PDL1 mAb immunotherapy in metastatic TNBC patients [113]. In addition, the conclusions from the KEYNOTE-522 and KEYNOTE-119 trials suggest a statistically significant benefit with a combination of anti-PD1 mAb with neoadjuvant therapy in TNBC regardless of PDL1 expression status [114,115]. However, it is important to note that multiple preclinical studies have suggested that chemotherapeutic agents induce the expression of immune inhibitory molecules, such as PDL1, CD47, and CD73, through DNA damage [116]. This could explain the application of immunotherapy in breast cancer patients regardless of the initial tumor expression of these immune inhibitory molecules.

The DDR pathway has been associated with the efficacy of anti-cancer therapy. Defects in the HRR pathway, which is one of the major DDR pathways, are shown to have a predictive application in the therapeutic success of PD-1 monoclonal antibodies (mAb) in metastatic castration-resistant prostate cancer [117]. Similarly, another DDR defect, a mismatch repair (MMR) pathway, could predict the positive outcome of ICI therapy in colorectal cancer [118]. Studies have shown that deficiencies in both these DDR pathways result in enhanced tumor mutational burden (TMB). Using targeted NGS to determine TMB, Wang et al. have shown that 22.3% of pan-cancer patients have enhanced HRR/DDR gene expression. Furthermore, 30.4% of these HRR/DDR+ patients showed TMB [119]. However, this study did not show any correlation between TMB and PDL1 expression. In contrast, a retrospective review of two melanoma studies, Checkmate 066 and Checkmate 067, showed that high TMB is associated with enhanced immunotherapeutic efficiency [120,121,122,123]. Mutations of HRR pathway genes can lead to the expression of immunogenic neo-antigens leading to effector CD4 and CD8 immune responses. However, there is no association between HRD and TIL frequency in breast tumor tissues. Further, a pooled analysis of five clinical trials showed no correlation between HRD status and either the expression of immune inhibitory molecules or TIL frequency [10]. Several clinical trials have initially concluded that a combination of DNA-damaging drugs with ICI-based immunotherapy was safe and possibly has a better therapeutic efficacy [124]. However, in-depth conclusions from several of these studies are still awaited.

Studies by You et al. have shown that patients with mutations in HRR genes had positive outcomes with anti-CTLA4 therapy over anti-PD-1/PD-L1 [125]. It is possible that factors other than TMB, such as tumor-infiltrating immune cell phenotype and antigen presentation capability, could also impact this higher efficacy of anti-CTLA4 therapy. As anti-CTLA4 is associated with higher systemic cytokine storm and cardiac events than anti-PD1/anti-PDL1, an analysis of HRR pathway genes might guide the selection of specific ICI agents [126]. Interestingly, the highest response to ICI blockade (ICB) was shown in tumors that had a higher immune cell infiltration [127]. Cellular biomarkers, like neutrophil-to-lymphocyte ratio, and molecular biomarkers, such as PD-L1 and LDH, are extensively used to predict the success of ICB [18]. Among all the predictors, the TMB rate, defined by a high number of somatic mutations, has been shown to have the best correlation with ICB therapeutic success. Studies by Le et al. have shown that there was a 53% objective radiographic response and 21% complete response in MMR-deficient cancers to pembrolizumab across 12 tumor types [128]. Based on these data, the FDA has approved the usage of ICB therapy in MMR-deficient tumors. Interestingly, a retrospective multivariate analysis determined that the presence of a family history of cancer in patients treated with anti-PD1/PDL1 demonstrated a better objective response rate (ORR) and median overall survival (OS) [129]. This is important in the context of germline mutations in HRR-pathway *BRAC1/2* genes, which have been strongly associated with the hereditary risk of breast cancer [130]. The IMpassion130 phase III study showed that anti-PDL1 plus paclitaxel-based chemotherapy in metastatic TNBC prolonged progression-free survival (PFS) along with a hazard ratio (HR) of 0.62 (*p* < 0.001) [96]. While correlation with the HRR pathway was not directly analyzed in this study, it is important to note that up to 35% of the TNBCs demonstrate BRAC1/2 mutations [131]. All these studies suggest a strong correlation between HRD and ICB success.

## 4. Impact of BRCAness on Early Breast Cancer Treatment

TNBC is shown to have the highest TMB among all breast cancer subtypes [132,133]. Further, 10–14% of TNBCs show germline BRCA1/2 mutations [134,135]. In early-stage TNBC, preoperative treatment with anthracyclines and taxane-based neoadjuvants is a well-accepted standard of care for down-staging, along with achieving pathologic complete response (PCR) in 30–40% of cases [136]. A possible addition of PARPis or platinum-based chemotherapy could further cumulatively enhance therapeutic efficacy. Initial randomized trials that studied the impact of adding platinum-based salts in TNBC patients, such as the GEICAM/2006-03 and CALB-B 40603 trials, failed to show its efficacy mainly because the patient inclusion criteria in these trials did not take into account BRCA status [137,138]. Conversely, cisplatin-based neoadjuvant studies in early-stage TNBC by Byrski et al., which included only patients with germline BRCA alteration, demonstrated a PCR of 61% [139]. Similarly, the GeparSixto and BrighTNess trials have also demonstrated beneficial outcomes with the inclusion of platinum-salt-based and/or PARPi therapy in the treatment of TNBC patients with BRCA mutations [140,141]. However, the cisplatin cohort in the INFORM trial, which included only germline-BRCA-altered patients with advanced-stage TNBC, did not show much efficacy [142]. The practice-changing landmark study, OlympiA trial, which specifically included early-stage breast cancer patients with germline BRCA1/2 mutations, clearly demonstrated that olaparib (PARPi) in an adjuvant setting had a significantly higher overall survival benefit (HR = 0.68, *p* = 0.009) [143]. Taken together, all these studies point out that early-stage breast cancer patients with HRD benefit from DNA-damaging agents (platinum-based) and/or PARPi-based adjuvant therapy. A comprehensive list of clinical trials exploring the role of DNA-damage-inducing agents in early-stage breast cancer patients with HRD is provided in Table 1.

An HRD score of >42 (as analyzed by Myriad Genetics, Inc, Salt Lake City, UT, USA) regardless of BRCA1/2 status was shown to be a better predictor of the efficacy of platinum- and PARPi-based neoadjuvant therapy in TNBC [10]. Interestingly, Davies et al. have proposed a lasso logistic regression model to develop six critically distinguishing mutational signatures enabling the identification of HRD (the authors termed it ‘HRDetect’). This has allowed the identification of functional BRCA1/2 deficiency (BRCAness) in addition to determining germline and somatic *BRCA1/2* mutations. The sensitivity and specificity of HRDetect were validated by whole genome sequencing. This has allowed the identification of BRCAness (including germline and somatic *BRCA* mutations) in 22% of breast cancer patients as against the 5–10% identified by the genomic-scar-based HRD score [144]. This includes epigenetic alterations in the promoter region of *BRCA1/2* along with genetic and/or epigenetic alterations in HRR pathway genes such as *RAD51C* and *PALB2* [145]. This gene mutational-signature-profile-based identification of BRCAness could allow a higher selectivity of patients who could respond to cisplatin and PARPi-based therapy. As there are more than 50 genes in the HRR pathway, several research groups have advocated for the usage of a multi-gene mutational profile to determine HRD over a comprehensive genomic scar score based on genomic instability markers [68]. 

The data from the GeparSixto and BrighTNess trials showed that TNBC patients with high HRD scores (>42) benefitted from the addition of carboplatin to their treatment [140,141]. Similarly, evidence from the PETREMAC study showed that TNBC patients on PARPis had an ORR of 56%, with the majority of responders having HRD-high status [88]. All these data clearly suggest that genomic instability is one of the best markers to predict the efficacy of PARPis and platinum-based adjuvant therapy. In contrast, a phase II randomized trial, TBCRC030, did not show the efficacy of platinum-based adjuvant therapy in TNBC patients with HRD scores both at a cut-off of >33 and at >42 [146]. The conclusions from other interesting studies, such as PEARLY (NCT02441933) and PARTNER (NCT03150576), are still awaited, and these may provide better evidence for a possible widespread application of HRD genetic testing in cancer treatment.

## 5. Impact of BRCAness on Metastatic Breast Cancer Treatment

There is relatively limited evidence on the role of the HRR pathway and the utility of DNA-damaging drugs (platinum-based and PARPis) in metastatic breast cancer. Studies by Tutt et al. have shown that carboplatin treatment in metastatic TNBC patients with germline *BRCA1/2* mutation was associated with improved ORR (68% vs. 33%, *p* = 0.03) and enhanced PFS (6.8 vs. 4.4 months, *p* = 0.002) [147]. The OlympiAD trial has shown that the response rates significantly improved with olaparib in metastatic breast cancer patients with germline *BRCA1/2* mutations [148]. Similarly, the ABRAZO and EMBRACA trials have shown therapeutic benefits with PARPis in metastatic breast cancer patients with germline BRCA1/2 mutations [149,150]. Furthermore, the BROCADE3 trial has shown that a combination of DNA-damaging drugs (platinum-based plus PARPis) improved median PFS in metastatic breast cancer patients with BRCA alterations [151]. However, none of the above studies utilized HRD scores to assess the potential genomic instability status in tumors. Future studies assessing the correlation between HRD score and DNA-damaging drugs will be helpful in designing neoadjuvant therapy in metastatic breast cancer patients. A comprehensive list of clinical trials exploring the role of DNA-damage-inducing agents in metastatic breast cancer patients with HRR mutations is provided in Table 2.

In the TNT trial, carboplatin showed no improvement in ORR (carboplatin 44.7% vs. docetaxel 39.6%, *p* = 0.67) in HRD-high metastatic breast cancer patients [152]. Similarly, studies by Zhao et al. showed that HRD status (or BRCAness), as determined by whole genome sequencing, positively correlated with the benefits of platinum-based therapy in metastatic breast cancer patients [153]. A more stringent sub-cohort analysis by Galland et al., where patients were classified into *BRCA*-mutated, *BRCA* WT HRD-high (HRD score > 42), and *BRCA* WT HRD-low (HRD score 33–41), showed that the *BRCA* WT HRD-high cohort did not benefit from the usage of platinum-based therapy [12]. However, some limited studies, such as the TBCRC 048 trial and the RUBY study, have suggested the efficacy of PARPis in metastatic breast cancer patients with mutations in HRR pathway genes (germline *PALB2* or somatic *BRCA1*/*2*) [65,154]. Interestingly, large-scale genomic characterization of metastatic breast cancer studies by Bertucci et al. found that a high HRD score was seen in the ER+/HER2- subtype of metastatic breast cancer over early-stage breast cancer [155]. These data warrant further studies to verify the potential application of HRD score as a marker for the therapeutic application of PARPis and platinum-based therapy in subtypes other than TNBC. 

Studies by Mao et al. demonstrated that tumors with HRD-high mutational signatures had high expression of immune checkpoint inhibitors (ICIs), namely, CTLA4 and PD1 [156]. Further, studies by Teo et al. suggested the potential role of HRR pathway gene mutation testing in determining the therapeutic benefit of immunotherapy in urothelial cancers [157]. However, currently, there is limited evidence on the potential impact of the combination of PARPis with immunotherapy. The results from current ongoing trials (NCT03025035) will shed light on the future combination of DNA-damaging drugs with ICIs along with the potential application of HRD score as a therapeutic marker to predict the efficacy of immunotherapy.

## 6. Conclusions

With the emergence of personalized medicine, HRD testing will be critical to determine the appropriate therapeutic subset profiles of breast cancer patients. Advances in genomics and bioinformatics have enabled the identification of specific HRD molecular alterations. While *BRCA1/2* mutations are the standard in clinical testing, other HRR pathway genes, such as *PALB2* and *RAD51C*, have potential applications in determining breast cancer treatment. However, there needs to be a greater consensus on genomic scar scoring (e.g., HRD score vs. COSMIC signature), and direct comparisons between various HRD testing tools should be performed. Given the utility of the HRR pathway in determining the success of PARPis, platinum-based chemotherapy, and ICB in breast cancer, BRCAness should be more frequently utilized in patient stratification. It is also important to reduce the financial burden on patients by appropriate advocacy for this testing through medical insurance coverage.

## Figures and Tables

**Table 1:** Clinical trials studying the interplay of DNA-damaging drugs and HRD in early breast cancer.

Intervention	Identifier#	Title	HRD Score	Status
Carboplatin plus background treatment	NCT01426880	Addition of Carboplatin to Neoadjuvant Therapy for Triple-negative and HER2-positive Early Breast Cancer	HRD ≥ 42: 70.5% tBRCA1/2mt: 29% gBRCA1/2mt: 20%	Completed
Cisplatin	NCT01630226	Cisplatin Monotherapy in the Treatment of BRCA1 Positive Breast Cancer Patients in Poland	gBRCA1/2mt: 100%	Unknown
Carboplatin + nanoparticle albumin-bound paclitaxel + vorinostat (HDAC inhibitor)	NCT00616967	Carboplatin and Nab-Paclitaxel with or without Vorinostat in Treating Women with Newly Diagnosed Operable Breast Cancer	HRD ≥ 42: 46%	Active; not recruiting
Carboplatin + Eribulin	NCT01372579	Carboplatin and Eribulin Mesylate in Triple-negative Breast Cancer Patients	HRD ≥ 42: 46% gBRCA1/2mt: 10%	Unknown
Cisplatin vs. Doxorubicin/Cyclophosphamide	NCT01670500	Cisplatin vs. Doxorubicin/Cyclophosphamide in BrCa	gBRCA1/2mt: 68%	Active; not recruiting
Cisplatin vs. Paclitaxel	NCT01982448	Cisplatin vs. Paclitaxel for Triple-negative Breast Cancer	HRD ≥ 33: 71%	Completed
Carboplatin + gemcitabine + iniparib	NCT00813956	A Phase 2 Study of Standard Chemotherapy Plus BSI-201 (a PARP Inhibitor) in the Neoadjuvant Treatment of Triple-negative Breast Cancer	gBRCA1/2mt: 24%	Completed
Paclitaxel vs. paclitaxel + veliparib + carboplatin	NCT01042379	I-SPY TRIAL: Neoadjuvant and Personalized Adaptive Novel Agents to Treat Breast Cancer	gBRCA1/2mt: 17%	Actively recruiting
Paclitaxel vs. paclitaxel + carboplatin vs. paclitaxel + carboplatin + veliparib	NCT02032277	A Study Evaluating Safety and Efficacy of the Addition of ABT-888 Plus Carboplatin Versus the Addition of Carboplatin to Standard Chemotherapy Versus Standard Chemotherapy in Subjects with Early-Stage Triple-negative Breast Cancer	HRD ≥ 42: 67% gBRCA1/2mt: 15%	Completed
Paclitaxel-olaparib vs. paclitaxel-carboplatin	NCT02789332	Assessing the Efficacy of Paclitaxel and Olaparib in Comparison to Paclitaxel/Carboplatin Followed by Epirubicin/Cyclophosphamide as Neoadjuvant Chemotherapy in Patients with HER2-negative Early Breast Cancer and Homologous Recombination Deficiency	g/tBRCA1/2mt: 56.2%	Completed
Talazoparib	NCT03499353	Talazoparib For Neoadjuvant Treatment of Germline BRCA1/2 Mutation Patients with Early Human Epidermal-Growth-Factor-Receptor-2-Negative Breast Cancer	gBRCA1mt: 42.1% gBRCA2mt: 10.5%	Terminated (Not due to safety concerns)
Talazoparib	NCT02282345	Talazoparib Before Standard Therapy in Treating Patients with Invasive, BRCA-Mutated Breast Cancer	gBRCA1mt: 78.8% gBRCA2mt: 21.2%	Completed
Olaparib	NCT02624973	Personalized Treatment of High-risk Mammary Cancer—the PETREMAC Trial	HRD: 34% gBRCA1/2mt: 14%	Active; not recruiting
Niraparib	NCT03329937	Study Evaluating the Antitumor Activity and Safety of Niraparib as Neoadjuvant Treatment in Participants with Breast Cancer	gBRCA1mt: 67% gBRCA2mt: 28%	Completed
Olaparib	NCT02032823	Olaparib as Adjuvant Treatment in Patients with Germline BRCA Mutated High-Risk HER2 Negative Primary Breast Cancer	gBRCA1mt: 72% gBRCA2mt: 27%	Active; not recruiting
Rucaparib	EudraCT 2014-003319-12	Window study of the PARP inhibitor rucaparib in patients with primary triple-negative or BRCA1/2-related breast cancer	HRD: 69% gBRCA1/2mt: 19%	Completed

**Table 2:** Clinical trials studying the interplay of DNA-damaging drugs and HRD in metastatic breast cancer.

Intervention	Identifier#	Title	HRD Score	Status
Cisplatin or carboplatin	NCT00483223	Platinum for Triple-negative Metastatic Breast Cancer and Evaluation of p63/p73 as a Biomarker of Response	HRD score (LOH: 12.68; LST: 5.11)	Completed
Carboplatin vs. Docetaxel	NCT00532727	Triple-negative Breast Cancer Trial (TNT)	gBRCA1/2 mt BRCA1 methylation HRD score ≥ 42	Unknown
Cisplatin	H14-00681-A019	Homologous Recombination Deficiency and Platinum-Based Therapy Outcomes in Advanced Breast Cancer	HRD score(WGS) 20%	Completed
Carboplatin or Cisplatin	N/A	Efficacy of platinum-based chemotherapy in metastatic breast cancer and HRD biomarkers: utility of exome sequencing	HRD score and COSMIC signature 3 (WES)	Completed
Carboplatin + Paclitaxel vs. Carboplatin + Paclitaxel + veliparib vs. Veliparib	NCT02163694	A Phase 3 Randomized, Placebo-controlled Trial of Carboplatin and Paclitaxel with or without Veliparib (ABT-888) in HER2-negative Metastatic or Locally Advanced Unresectable BRCA-associated Breast Cancer	gBRCA1/2 mt	Active; not recruiting
Olaparib	NCT02000622	Assessment of the Efficacy and Safety of Olaparib Monotherapy Versus Physicians’ Choice Chemotherapy in the Treatment of Metastatic Breast Cancer Patients with Germline BRCA1/2 Mutations	gBRCA1/2 mt	Active; not recruiting
Talazoparib	NCT01945775	A Study Evaluating Talazoparib (BMN 673), a PARP Inhibitor, in Advanced and/or Metastatic Breast Cancer Patients with BRCA Mutation	gBRCA1/2 mt	Completed
Niraparib + Pembrolizumab	NCT02657889	Niraparib in Combination with Pembrolizumab in Patients with Triple-negative Breast Cancer or Ovarian Cancer	gBRCA1/2 mt	Completed
Olaparib + durvalumab	NCT02734004	A Phase I/II Study of MEDI4736 in Combination with Olaparib in Patients with Advanced Solid Tumors	gBRCA1/2 mt	Active; not recruiting
Olaparib	NCT03344965	Olaparib In Metastatic Breast Cancer	HRR pathway gene mutations	Recruiting
Rucaparib	NCT02505048	A Study to Assess the Efficacy of Rucaparib in Metastatic Breast Cancer Patients with a BRCAness Genomic Signature	LOH score or HRR pathway gene mutations	Completed
Talazoparib	NCT02401347	Phase II Trial of Talazoparib in BRCA1/2 Wild-type HER2-negative Breast Cancer and Other Solid Tumors	BRCA WT with HRR pathway gene mutations	Completed
Palbociclib + Olaparib + Fulvestrant	NCT03685331	HOPE: Olaparib, Palbociclib, and Fulvestrant in Patients with BRCA-Mutation-associated, HR+, HER2-metastatic Breast Cancer	gBRCA1/2 mt	Active; not recruiting
Durvalumab + Olaparib + Fulvestrant	NCT04053322	Durvalumab, with Olaparib and Fulvestrant in Advanced ER+, HER2- Breast Cancer Patients	gBRCA1/2 mt and HRR pathway gene mutations	Active; not recruiting
Pembrolizumab + Olaparib	NCT03025035	Pembrolizumab in Combination with Olaparib in Advanced BRCA-mutated or HDR-defect Breast Cancer	gBRCA1/2 mt and HRR pathway gene mutations	Recruiting
Niraparib, anti-TIM3, bevacizumab, and platinum-based doublet chemotherapy + anti-PD-1	NCT03307785	Study of Niraparib, TSR-022, Bevacizumab, and Platinum-Based Doublet Chemotherapy in Combination with TSR-042	HRD not a criterion	Active; not recruiting
Talazoparib + avelumab	NCT03565991	Javelin BRCA/ATM: Avelumab Plus Talazoparib in Patients with BRCA or ATM Mutant Solid Tumors	BRCA or ATM mutations	Not Recruiting (study continued as NCT05059522)
Rucaparib + atezolizumab	NCT03101280	A Combination Study of Rucaparib and Atezolizumab in Participants with Advanced Gynecologic Cancers and Triple-negative Breast Cancer	LOH or HRR pathway gene mutations	Completed
Olaparib + durvalumab	NCT02484404	Phase I/II Study of the Anti-Programmed Death Ligand-1 Durvalumab Antibody (MEDI4736) in Combination with Olaparib and/or Cediranib for Advanced Solid Tumors and Advanced or Recurrent Ovarian, Triple-negative Breast, Lung, Prostate, and Colorectal Cancers	gBRCA1/2 mt	Recruiting
Olaparib + durvalumab + copanlisib	NCT03842228	Testing the Combination of the Anti-cancer Drugs Copanlisib, Olaparib, and MEDI4736 (Durvalumab) in Patients with Advanced Solid Tumors with Selected Mutations	HRR pathway gene mutations	Recruiting
Olaparib + durvalumab	NCT03167619	Phase II Multicenter Study of Durvalumab and Olaparib in Platinum tReated Advanced Triple-negative Breast Cancer (DORA)	HRD not a criterion	Completed
